# Rare Case of Peripheral Myxofibroma of Anterior Maxillary Gingiva: A Diagnostic Challenge

**DOI:** 10.7759/cureus.45669

**Published:** 2023-09-21

**Authors:** Theodoros Lillis, Vasileios Zisis, Ioannis Fotopoulos, Helena Iordanidou, Eleftherios Anagnostou, Dimitrios Andreadis, Athanasios Poulopoulos, Nikolaos Dabarakis

**Affiliations:** 1 Dentoalveolar Surgery, Implantology and Oral Radiology, School of Dentistry, Faculty of Health Sciences, Aristotle University of Thessaloniki, Thessaloniki, GRC; 2 Oral Medicine/Pathology, School of Dentistry, Faculty of Health Sciences, Aristotle University of Thessaloniki, Thessaloniki, GRC

**Keywords:** maxillary anterior region, gingival lesion, odontogenic tumour, odontogenic, myxofibroma

## Abstract

The myxofibroma (MF) constitutes an uncommon, non-malignant, odontogenic neoplasm with potential mesenchymal derivation. The occurrence rate of this particular tumor is estimated to be around 0.05 new cases per million individuals annually. MFs exhibit a higher incidence rate within the age range of 10 to 30 years. The prevalence of these tumors is higher among the female population, with a predominant localization in the mandible, specifically in the posterior region. A female patient, 66 years old, was referred to the Department of Oral Surgery, Surgical Implantology and Radiology, Thessaloniki, Greece, complaining of a tumorous lesion in the anterior area of the maxilla and mild pain. Clinically, a solid in palpation lobulated tumor, covered by normal coloured mucosa was observed at the left upper incisor. After the excisional biopsy, the microscopic appearance of abundant fibromyxoid stroma, in particular, myxoid stroma intermingled with collagenous tissue, covered by stratified squamous epithelium, suggested the diagnosis of peripheral myxofibroma. During a 2-year follow-up, no recurrence was referred. This case illustrates the necessity of proper differential diagnosis of every tumorous lesion of the gingiva and of using the histopathological examination.

## Introduction

The myxofibroma (MF) constitutes an uncommon, non-malignant, neoplasm with potential mesenchymal derivation that was initially documented by Virchow in 1863 [1]. The peripheral myxofibroma constitutes a benign entity, of ectomesenchymal origin, which most commonly affects adult female patients in the anterior maxilla [2]. MFs are characterized by the presence of abundant collagen fibers distributed throughout a myxoid stroma [3]. A comprehensive literature review in 2015 reported a total of 24 documented instances of MFs [4]. The occurrence rate of this particular tumor is estimated to be approximately 0.05 new cases per million individuals annually [5]. MFs exhibit a higher incidence rate within the age range of 10 to 30 years [6]. The youngest patient to be recorded was 8 years old at the time of the diagnosis [7]. The prevalence of these tumors is higher among the female population, with a predominant localization in the mandible, specifically in the posterior region [8]. However, it is also supported that MF is equally distributed between males and females and that both maxilla and mandible are equally involved [9-10]. Small central MFs typically do not exhibit symptoms and are commonly detected through routine radiographic examinations. Conversely, larger central MFs are frequently characterized by painless jaw expansion and the potential for cortical plate perforation. Instances of facial deformity and maxillary sinus involvement have been infrequently documented in the literature [8,11]. Histologically, MFs are characterized by a substantial quantity of intercellular substance that is abundant in acid mucopolysaccharides. This intercellular substance is composed of loose myxomatous connective tissue, as well as fibroblasts and myofibroblasts [4]. The presence of dispersed patches of trabeculae of woven bone and capillaries is typically observed within the lesion [4]. From a radiographic perspective, the appearance of a central MF is characterized by the presence of a radiolucent area that can be either unilocular or multilocular, exhibiting irregular or scalloped margins. This condition has the potential to displace or induce resorption of the roots of neighboring teeth [12]. There is a lack of consensus regarding the optimal approach to management strategy. The available treatment options encompass a range of approaches, including a conservative strategy involving the removal of the lesion through enucleation and subsequent cavity curettage, as well as more aggressive radical surgical interventions. Certain authors propose the recommendation of enlarging surgical margins to a minimum of 1.5 cm surrounding the neoplasm [8]. The excision of a tumor may be correlated with the extraction of teeth that are potentially associated with it. The conservative approach appears to exhibit a correlation with a higher rate of recurrence, potentially reaching up to 25%. Recurrence typically manifests within the initial two-year period following the initial treatment [4]. MFs exhibit a recurrence rate ranging from 25% to 43% [8]. A plausible explanation is that since MFs may originate from the periodontal ligament of the neighboring teeth, they function as a source of the cells involved in the MF pathogenesis. The objective of this study is to present a rare case of peripheral MF, located solely on the gingiva of #21 and conduct a review on the topics of epidemiology, clinical and radiographic characteristics, treatment modalities, and rates of recurrence in patients with MF.

## Case presentation

**Figure 1:**
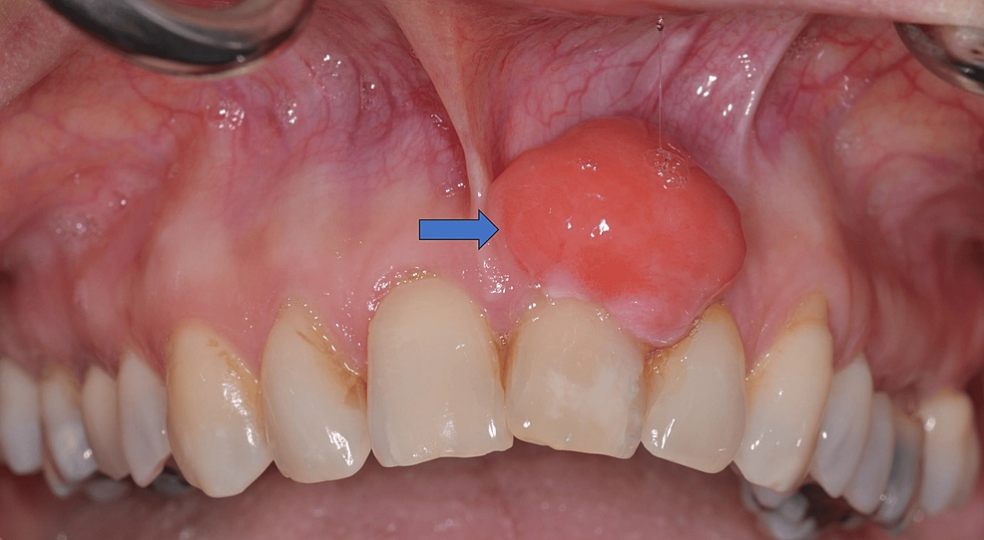
Initial clinical appearance of the lesion (blue arrow).

A female patient, 66 years old, came to the Department of Oral Surgery, Surgical Implantology and Radiology, School of Dentistry, Aristotle University of Thessaloniki, Greece, complaining of a tumorous lesion in the anterior area of the maxilla and mild pain due to food impaction in the interdental area. Before the examination, the patient provided written informed consent. This form was approved by the School of Dentistry, Aristotle University of Thessaloniki, and was in accordance with the Helsinki Declaration for research and patient ethics. Subsequently, the patient was examined thoroughly and referred for further examination to the Department of Oral Medicine and Pathology, School of Dentistry, Aristotle University of Thessaloniki, Greece. Clinically, a sessile, lobulated movable solid in palpation tumor was observed, covered by normal to erythematous mucosa, which derived from the gingiva of #21. The differential diagnosis included common entities such as fibroma, peripheral ossifying fibroma, giant cell tumor, and less common entities such as myxoma, ameloblastoma, ameloblastic fibroma, odontogenic fibroma or odontogenic keratocystic tumor. Figure 1 displays the initial clinical appearance of the lesion.

**Figure 2:**
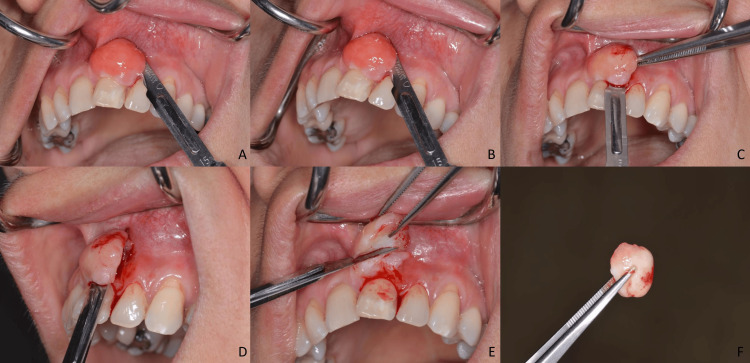
Display of the stepwise surgical excision of the tumor. A-E: Gradual excision of the tumor. F: Display of the removed tumor.

The surgical procedure took place in the Department of Dentoalveolar Surgery, Surgical Implantology and Radiology and was carried through in a conservative fashion. A scalpel #15 was used for the excision and precautions were taken, to avoid any unnecessary removal of soft tissue, which could undoubtedly lead to gingival recession and further aesthetic complications for the patient. Subsequently, sutures were placed, and the surgical area was covered with surgical cement. The stepwise surgical procedure is displayed in Figure 2.

**Figure 3:**
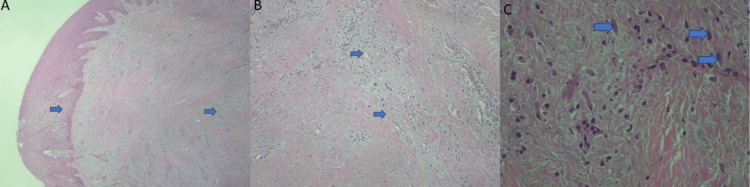
Histopathological examination of the tissue specimen. A: Abundant fibromyxoid stroma is covered by stratified squamous epithelium, which presents hyperplasia and spongiosis (blue arrows) (H-E X40 magnification). B: Myxoid stroma was intermingled with collagenous tissue with numerous inflammatory cells (blue arrows) (H-E X100 magnification). C: High-power view shows scattered spindle-shaped cells, within the myxoid stroma, admixed with lymphocytes and plasma cells (blue arrows). Nuclear atypia or mitoses are not identified (H-E X400 magnification).

The tissue specimen was preserved in formaldehyde and submitted to histopathological examination in the Department of Oral Medicine/Pathology of the same Institution. Microscopically, an abundant fibromyxoid stroma, covered by stratified squamous epithelium, which presents hyperplasia and spongiosis was seen. The myxoid stroma was intermingled with collagenous tissue with numerous inflammatory cells. Scattered spindle-shaped cells, within the myxoid stroma, admixed with lymphocytes and plasma cells were also noticed. Nuclear atypia or mitoses were not identified. Therefore, the diagnosis of peripheral MF was established (Figure 3).

**Figure 4:**
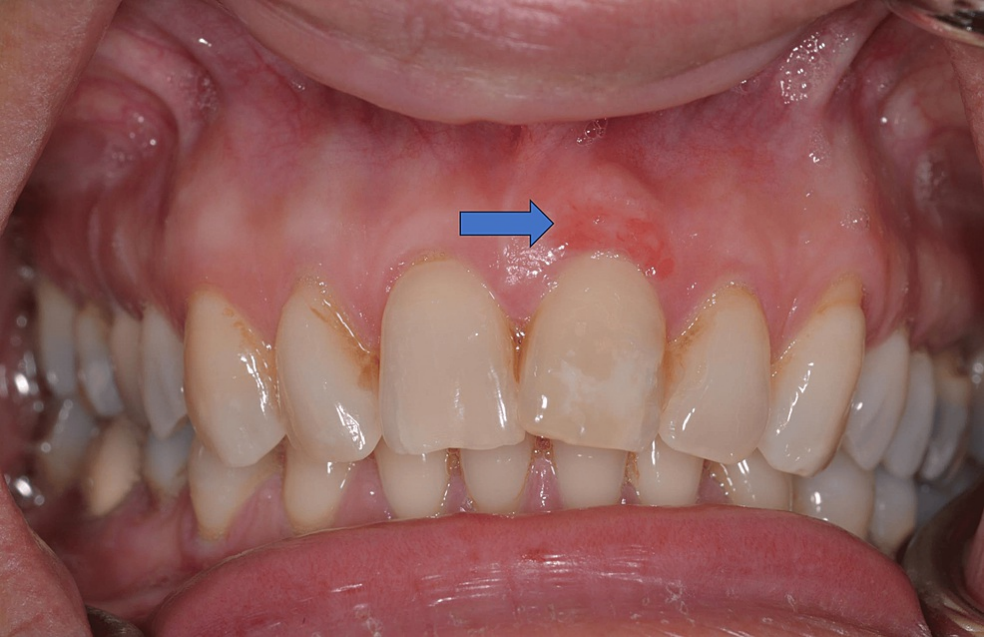
An erythematous area remains, two weeks after the excision of the tumor.

The recall of the patient took place two weeks later where the successful wound closure was observed and only an erythematous area was still present, due to the neovascularization process (Figure 4).

During a 2-year follow-up, no recurrence was referred.

## Discussion

Central type of MFs possess a tendency for expansion, primarily manifest in the jaws, with a significantly high predilection for this anatomical region. The mandible exhibits a higher incidence of involvement compared to the maxilla [13-15], with a particular preference for the posterior region in both jaws [3]. In our case presentation, the anterior part of the maxilla was affected. A recent case where the MF originates from the periodontal ligament from the posterior maxillary region [16], is reported in the literature. The first record of MF, originating from the periodontal ligament, was written in 1975 [17]. The majority of cases documented in the literature were identified during the age range spanning from the second to the fourth decades of life, with a notable concentration of diagnoses occurring during the third decade [3].

The majority of MFs exhibit no symptoms, although a small number of patients have reported experiencing escalating pain due to the invasion of adjacent structures [4]. Evidently, individuals afflicted with tumors situated in the posterior region exhibited delayed diagnoses and larger lesions in comparison to those affected by tumors located in the anterior region. This phenomenon can likely be attributed to the heightened visibility of disfigurement when the lesions are situated in the anterior region such as in our case, where the patient came to the clinic, exclusively due to aesthetics-related concerns. In a manner consistent with other odontogenic tumors, such as ameloblastoma, various descriptors such as "soap bubbles," "ground glass," or "tennis racquet strings" have been employed to characterize the radiographic manifestations of these lesions. Given the potential for varying interpretations and the inherent confusion surrounding these descriptors, it may be necessary to provide a more precise definition of the radiological characteristics of myxomas and MFs. This would serve the purpose of establishing more objective diagnostic criteria. Based on the available literature, the clinical and radiographic differential diagnoses for MFs encompass various conditions such as fibroma, peripheral ossifying fibroma, giant cell tumor, ameloblastoma, ameloblastic fibroma, odontogenic fibroma, odontogenic keratocystic tumor, central hemangioma, aneurysmal bone cyst, and less common entities like desmoplastic fibroma [4]. The macroscopic characteristics of the surgical specimen, characterized by a firm and fibrous texture, prompted the surgeons to consider the possibility of a myxomatous lesion [3].

Regarding the remaining odontogenic tumors, the conclusive diagnosis of MFs relies on the assessment of histopathological characteristics. If the size of the lesions is substantial, it may be necessary to conduct a biopsy in order to determine the characteristics of the tumor and develop an appropriate therapeutic strategy. The preferred treatment modality, as reported in the literature, is surgical excision [18]. There is currently a lack of consensus regarding the optimal extent of surgical margins. The scarcity of MF cases significantly hinders the acquisition of dependable data regarding prognosis following various surgical methodologies. Conservative surgery, characterized by the enucleation of the lesion and curettage of the residual tissue, presents certain advantages in comparison to more radical approaches, such as tumor resection along with adjacent tissues [4]. There are several advantages associated with this approach, including a decrease in morbidity, the potential to avoid reconstructive surgery, a shorter duration of hospitalization, minimized disruptions to facial growth in pediatric patients, and reduced financial burden. However, certain authors recommend radical treatment, such as en-bloc resection, due to the distinctive features of MFs, including their locally aggressive behavior, potential for significant size, and tendency to recur [5,18]. Recurrence is commonly linked to the phenomenon of local invasion into the cancellous bone that extends beyond the radiographically detectable margins, occurring in the absence of tumor encapsulation. It has been observed that a more intensive treatment approach, involving the performance of either a partial or complete segmental bone resection with tumor-free margins measuring 1.5 cm, can potentially lead to a decrease in the rate of recurrence. This particular treatment option may be considered advantageous in the maxilla, when the lesion is huge, due to its proximity to the maxillary sinus, zygoma, and lower portion of the orbital cavity, which can pose a critical factor in the event of recurrence [8]. The decision to pursue conservative surgery is further substantiated by the lack of documented instances of malignant transformation in MFs, as well as the relatively low recurrence rate observed in certain case series following conservative management [12]. It is recommended to conduct postoperative monitoring of the patient for a minimum duration of three years, as studies indicate a heightened likelihood of recurrence within this timeframe [8]. The appearance of synchronous or metachronous odontogenic myxofibromas, in patients affected by rare, clinical entities, which may be characterized by genetic predisposition, supports the notion of a possible genetic background underlying the manifestation of MF. In particular, MF with angiosarcoma, MF with tuberous sclerosis, and MF with myasthenia gravis are reported in the literature [1,19,20]. In summary, based on the aforementioned information on MFs, peripheral MFs located in the oral mucosal soft tissues are extremely rare. The decision for a conservative excision was taken, both due to the absence of bone involvement and to avoid any aesthetic complications regarding gingival recession on the #21. According to the literature, regular check-ups are considered to be of crucial importance, to prevent any major recurrence, especially during the span of the first three years, postoperatively.

## Conclusions

The initial differential diagnosis included common entities such as fibroma, peripheral ossifying fibroma, giant cell tumor, and less common entities such as myxoma, ameloblastoma, ameloblastic fibroma, odontogenic fibroma or odontogenic keratocystic tumor. The best case scenario would be the fibroma, but since 100% accuracy and certainty are not possible based solely on clinical features, any tissues, after removal, should be submitted to histopathological examination, regardless of how benign they appear to be. The peripheral location of the MF allowed for a conservative excision, avoiding any further functional or aesthetic complications. During a 2-year follow-up, no recurrence was referred.
